# Coliphages as viral indicators of sanitary significance for drinking water

**DOI:** 10.3389/fmicb.2022.941532

**Published:** 2022-07-26

**Authors:** Suniti Singh, Robert Pitchers, Francis Hassard

**Affiliations:** ^1^Cranfield Water Science Institute, Cranfield University, Bedford, United Kingdom; ^2^Water Research Centre, Swindon, United Kingdom; ^3^Institute for Nanotechnology and Water Sustainability, University of South Africa, Johannesburg, South Africa

**Keywords:** coliphage, somatic coliphage, drinking water quality, online monitoring, drinking water treatment, F^+^ coliphage

## Abstract

Coliphages are virus that infect coliform bacteria and are used in aquatic systems for risk assessment for human enteric viruses. This mini-review appraises the types and sources of coliphage and their fate and behavior in source waters and engineered drinking water treatment systems. Somatic (cell wall infection) and F^+^ (male specific) coliphages are abundant in drinking water sources and are used as indicators of fecal contamination. Coliphage abundances do not consistently correlate to human enteric virus abundance, but they suitably reflect the risks of exposure to human enteric viruses. Coliphages have highly variable surface characteristics with respect to morphology, size, charge, isoelectric point, and hydrophobicity which together interact to govern partitioning and removal characteristics during water treatment. The groups somatic and F^+^ coliphages are valuable for investigating the virus elimination during water treatment steps and as indicators for viral water quality assessment. Strain level analyses (e.g., Qβ or GA-like) provide more information about specific sources of viral pollution but are impractical for routine monitoring. Consistent links between rapid online monitoring tools (e.g., turbidity, particle counters, and flow cytometry) and phages in drinking water have yet to be established but are recommended as a future area of research activity. This could enable the real-time monitoring of virus and improve the process understanding during transient operational events. Exciting future prospects for the use of coliphages in aquatic microbiology are also discussed based on current scientific evidence and practical needs.

## Coliphage characteristics

Phages are the most widely distributed and abundant biological forms on Earth estimated at ~ 10^31^ particles in the biosphere (Hendrix et al., 1999; Comeau et al., 2008; Mushegian, 2020). Phages are part of a complex microbial ecosystem and exist either as free-floating infectious particles in environmental matrices or within a bacteria or associated directly/indirectly to particles (Clokie et al., 2011; Zimmerman et al., 2020). Phages are obligate parasites of prokaryotes and replicated by members of two domains of cellular life—bacteria and archaea, with some evidence of interactions with eukaryotic organisms through effects to their microbiome or to their bacterial pathogens (Sime-Ngando, 2014; Putra and Lyrawati, 2020). The coliphages are a specific group of bacteriophages that infect coliforms (including *Escherichia coli*) and other closely related bacteria that are present in human and animal gut microbiomes.

**Figure 1:**
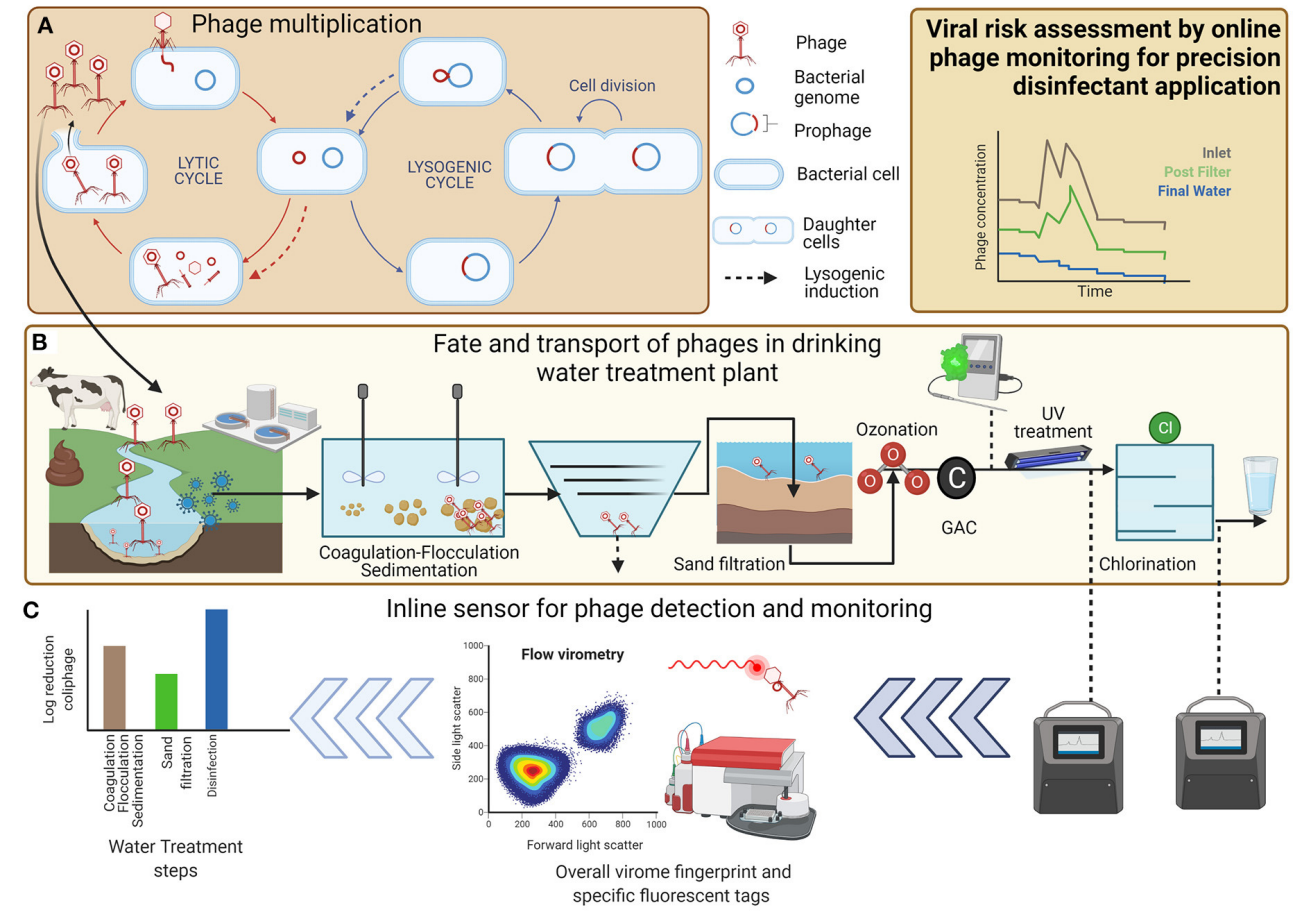
Schematic representing coliphages as viral indicators of sanitary significance for drinking water. Each panel represents a different example of this field. **(A)** Phage multiplication in environmental water matrices, **(B)** fate and transport of phages in drinking water treatment plant, and **(C)** inline sensors for phage detection and monitoring for disinfection optimization and risk assessment.

The coliphages are a diverse group of phage from several families and therefore consensus genomic sequences do not exist, which prevents design of universal primer sets limiting the use of qPCR for quantification of coliphage. Instead, they are classified into diverse taxonomic groups based on their *replication mechanism, mode of infecting hosts, morphological characteristics*, and *genomic content* (Table 1). Coliphages can be divided in two groups, virulent or temperate, which depends on their *replication mechanism* in host cells (Grabow, 2001). Virulent phages follow an obligate lytic cycle, whereas temperate phages undergo lysogenic cycle from where they can switch to a “lytic” or “chronic” cycle (Figure 1A). Coliphages infect host cells by first adsorbing to the host cell and injecting genetic material (DNA or RNA). Next, the coliphage nucleic material circularizes and enters lytic (virulent phage), lysogenic (temperate phage), or chronic cycle (temperate phage) (Figure 1A). In a lytic cycle, coliphages replicate in their host cells to synthesize new coliphage DNA or RNA and proteins which are assembled into phage virions. Subsequently, large numbers of coliphage virions can be released to the environment during each lytic cycle *via* lysis-protein-mediated rupture of the host cell wall. There are similarities between the lytic cycle and the lysogenic cycle. In the lysogenic cycle, coliphage attaches to the host cell and injects its genetic material into the bacteria host. During these processes, the coliphage DNA stably integrates into the chromosome of host bacterium forming a *prophage*. This entity is not infectious or lethal and replicates alongside and within the host DNA, increasing its titer with each prokaryotic replication cycle. Intermittently, an environmental cue will trigger the *prophage* to excise from the bacterial chromosome to become lytic. This marks a shift in the physiological state of host cells from lysogenic to lytic: a process which is termed *induction*. After *induction*, host cells are lysed, producing new phage virions (Figure 1A). The lysis of host cells expels intracellular components and cell debris into the surrounding environment and contributes to organic loading in waters. Thus, lytic phages (alongside higher organism grazing) have a key role in driving biogeochemical nutrient cycling and availability by structuring the population dynamics of their hosts in most aquatic systems (Clokie et al., 2011; Sime-Ngando, 2014). Another aspect of phage biology is the so-called chronic mode. This mode is exhibited by some archaeal phages but has not been widely reported in coliform bacteria. In chronic mode, the cell grows at a slower rate as new virions are continuously produced and excreted at low levels, despite the infected host cell remaining intact and viable (Howard-Varona et al., 2017).

**Table 1:** Characteristics of major groups of bacteriophages and their representative phage species.

**Family**	**Phage genus**	**Representative species**	**Type**	**Genogroup based on serotypes**	**Nucleic acid**	**Virion shape**	**Virion diameter (nm)**	**Tail**	**Measured pI**
*Levivirales*	*Fiersviridae (earlier Leviviridae)*	*Levivirus*	*Escherichia virus MS2*	F-specific RNA phage	Genogroup I	ssRNA, linear genome	icosahedral	26	No tail	2–4, 9.04^A^
			*Escherichia virus BZ13*		Genogroup II	ssRNA, linear genome	icosahedral	26	No tail	2.1–2.3
		*Allolevirus*	*Escherichia virus Qbeta (Qβ)*		genogroup III	ssRNA, linear genome	icosahedral	26	No tail	2–4, 5.3, 2–7, 1.9
			*Enterobacteria phage SP*		Genogroup IV	ssRNA, linear genome	icosahedral	26	No tail	2.1–2.6, 6.37^A^
*Tubulavirales*	*Inoviridae*	*Inovirus*	*Enterobacteria virus M13*	F-specific DNA phage	Not applicable	ss DNA, circular genome	filamentous	6, (1,000–2,000 nm long)	No tail, flexible filaments	4.05, 7.33^#^
*Kalamavirales*	*Tectiviridae*	*Alphatectivirus*	*Salmonella virus PRD1*	Somatic	Not applicable	ds DNA, linear genome	icosahedral	62	Pseudotail	3–4, 6.82^A^
*Caudovirales*	*Myoviridae*	*T4virus*	*Enterobacteria virus T4*			ds DNA, linear genome	elongated icosahedral	90, (200 nm long)	Long contractile tail	2, 4–5, 6.53^A^
	*Siphoviridae*	*Lambdavirus*	*Enterobacteria virus lambda*			ds DNA, linear genome	icosahedral	62	Long non-contractile tail	3.8, 7.04^#^
	*Podoviridae*	*T7 virus*	*Enterobacteria virus T7*			ds DNA, linear genome	icosahedral	55	Short non-contractile tail	6.98^A^
*Petitvirales*	*Microviridae*	*phix174*	*Escherichia virus phiX174*			ss DNA, circular genome	icosahedral	25	No tail	6–7.4, 7.66^A^

# Average calculated pI for phage proteome.

Activation of the lytic-lysogenic switch occurs either spontaneously or requires stimulation by natural or anthropogenic inductors. Induction can be triggered by exposure of bacterial host (containing prophage) to stressed conditions, such as UV, low nutrient conditions, a change to the bacterial trophic status (i.e., reduced competitors due to antibiotic), or replication inhibitors (mitomycin C); or through inactivation of a phage repressor. This process inevitably contributes to the coliphage loads in the surrounding environmental matrices. This aspect of (coli)phage biology offers a potential challenge to its utility as a viral indicator—as its abundance can be linked to its environment, e.g., solar irradiation in catchment or UV dose in treatment processes, rather than the fecal load exclusively. The impact of lysogeny on coliphage loading in an aquatic system is reviewed below.

Lysogeny and its contribution to phage abundance in aquatic matrices have been studied extensively. For example, the induction of lysogenic bacteria (lysogens) from tropical marine waters by mitomycin method contributed to 4–27% of the total marine phage concentrations (Ashy and Agustí, 2020). Exposure to surface ambient ultraviolet radiation levels induces lysogeny in bacteria within lake waters (Maranger et al., 2002). Solar radiation or hydrogen peroxide stress induces lysogeny and accounts for up to 29–63 and 47–53%, respectively, of phages produced by induction (Weinbauer and Suttle, 1999); however, the lysogenic contribution is low in marine (0.8–11%) and riverine bacteria (1%) (Muniesa and Jofre, 2007), suggesting total phage concentrations are governed by other factors than *in situ* lysogenic contribution, a finding which has been replicated in raw sewage and river waters (Casjens, 2003; Jofre, 2009). Therefore, the majority of coliphage loading, i.e., *plaque forming units* (PFU), are contributed from lytic coliphages generated within the gut of animals and to a lesser extent in the environment.

The PFU is an indicative measure of infective phage numbers. A single PFU can manifest from simultaneous infection initiated by a single- or multiple-phage particles (containing numerous copies of phage). Therefore, theoretically, one PFU is not always equivalent to one infective phage particle. However, from a practical perspective, the plaque numbers correlate well to absolute numbers of phage quantified via other methods (e.g., PCR-based approaches; Rose et al., 1997). However, what is less well-established is the influence of water physicochemistry on coliphage enumeration. The presence of natural organic and inorganic compounds can result in phage complexes to form. Key water treatment processes such as coagulation/flocculation aggregate particles and remove substantial amounts of virus from water. However, the aggregative effect could artificially reduce the quantification of phages (as multiple infective phages could present single plaque). In addition, Matsushita et al. (2011) identified that PFU infectivity assays were particularly sensitive to coagulants which impacted both the infectivity and the aggregative properties of coliphages compared to qPCR methods. Determining whether water treatments (e.g., coagulation) can impact viability (infectivity) via plaque assay or just result in false low numbers due to aggregation is critical, considering the role of infectious virus dose on human enteric/respiratory disease response. This is particularly important for understudied areas of water treatment with respect to virus and their indicators.

Coliphages can also be classified based on their “*mode of infection a host cell*” into somatic and F^+^ coliphages (also called male-specific or F-specific coliphages). Generally, individual coliphage strain infects specific *E. coli* strains by attaching to lipopolysaccharide or protein receptors in the cell wall and may lyse the host cell in within 20 min of infection (De Paepe and Taddei, 2006; Stone et al., 2019). They produce plaques of diverse size and morphology and can be useful for distinguishing different types of phages. Coliphage-host infection dynamics is strain specific (Molina et al., 2020), and as such model somatic phages are often used. One commonly applied coliphage during laboratory and pilot-scale experiments is phiX174 (ΦX174) which is host specific to *E. coli* ATCC 13706 (strain C) and PC 0886. In contrast, some other somatic coliphages may multiply in other hosts such as the *Enterobacteriaceae*. Of these, the two most commonly found species are *Shigellla* sp. and *Klebsiella pneumoniae* (Leclerc et al., 2000; Goodridge et al., 2003; Muniesa et al., 2003). Four phage families, *Myoviridae, Siphoviridae, Podoviridae*, and *Microviridae*, include strains which are considered to be somatic coliphages. The *Microviridae* group infects a diverse range of hosts such as *Enterobacteria, Bdellovibrio, Chlamydia*, and *Siroplasma*. For the *Myoviridae* group, principal hosts are *Enterobacteria, Bacillus*, and *Halobacterium*. For the *Siphoviridae* group, *Enterobacteria, Mycobacterium*, and *Lactococcus* are the major host groups. For the *Podoviridae* group, *Enterobacteria* and *Bacillus* are the main host groups (Lee, 2009). The tailed bacteriophages (order *Caudovirales*, including families *Myoviridae, Siphoviridae, Podoviridae*, and *Microviridae*) constitute 96% of all known phages (Ackermann, 1998). Of these, there are at least 150 “species” of *Caudovirales* phages (International Committee on Taxonomy of Viruses [ICTV]) which infect the genus *Escherichia*. Sequencing technologies are driving new phage discoveries which in turn is shedding light on the ecology of these entities (Korf et al., 2019) and the development of phage analysis tools, e.g., VIrus Classification and Tree building Online Resource (VICTOR) is aiding efforts for rapid and systematic phage classification (Meier-Kolthoff and Göker, 2017).

F^+^ coliphages are either single-stranded (ss) DNA or RNA viruses that are infectious to bacteria possessing fertility (F)-plasmid and infect their host through the F-pili (Jones et al., 2017). Of the F^+^ coliphages, the single-stranded RNA phages (FRNAPH) have been subclassified further into four serologically and phylogenetically distinct genogroups (designated GI, GII, GIII, and GIV). These FRNAPH genogroups differ in their tail morphologies (Table 1). Subsequently, several types of quantitative (reverse) PCR assays have been developed for rapid, sensitive, and specific detection of each F+RNA coliphage genogroup by targeting different conserved genes including the RNA replicases, maturase, capsid (coat protein), β-chain, or assembly genes (Jofre et al., 2016). The specific coliphage genogroups can assist in discrimination of the sources of fecal contamination via apportioning the load originating from humans and other animals—offering application for pollution tracking in catchments. FRNAPH strains MS2 (GI), GA (GII), Qβ (GIII), and SP (GIV) are widely used as representatives of their genogroups. There is some concern about the specificity of these genogroups to *E. coli* as the F-plasmid is transferable from/to *E. coli* and other related *Enterobacteria*. The transfer of the PCR target regions used in detecting FRNAPH genogroups (RNA replicases, maturase, capsid, or assembly genes) through the F-plasmid may confound quantification of FRNAPH genogroups, especially as a single bacterium is likely to contain numerous copies of the plasmid. For example, F^+^ coliphages may multiply in coliforms, including not only *E. coli* but also *Salmonella, Shigella, Bacteroides, Caulobacter, Pseudomonas*, and *Acinetobacter* providing an appropriate pili expressed on the bacterial surface (Leclerc et al., 2000; Cann, 2001; Virolle et al., 2020). The fragments of genetic material may be transferred along with F-plasmid, resulting in horizontal gene transfer, thereby transmitting genes (pathogenicity, metabolic properties, or antimicrobial resistance linked) among the bacteria across the water distribution network (Maganha de Almeida Kumlien et al., 2021). Few studies have reported the use of strain level monitoring in microbial source tracking (Hartard et al., 2016; Fauvel et al., 2017; Lee et al., 2019; Hata et al., 2021) as differentiating the F^+^ strains remains challenging and labor intensive.

## Fate and transport of coliphages in water used for human consumption

Coliphages are found in aquatic systems which are impacted by fecal contamination, including most surface and ground water drinking water sources, demonstrating region- and season-specific variation in abundance (Nappier et al., 2019). Coliphages are shed in high numbers in feces, with numbers dependent on the type of coliphage, animal host, and size and frequency of defecation events. There is some evidence of coliphages replicating in bacteria naturally present within surface water environments (Figure 1; Hassard et al., 2016), although low specific densities of host or phage are unlikely to permit coliphage replication in most aquatic environments (Muniesa and Jofre, 2004). Therefore, replication of coliphage in natural waters does not contribute a detectable increase in the numbers of somatic coliphages (Jofre et al., 2016). For some coliphages, e.g., F^+^ coliphages, the F+ pili is not readily produced by the host at <25°C; therefore, phage attachment and replication are unlikely in temperate water sources (Franke et al., 2009). Thus, the fate and behavior of coliphages are useful to assess the ability of water treatment to eliminate human enteric viruses and assess degree of contamination of surface and groundwaters (Jofre et al., 2016). Coliphages are considered in ambient water quality regulations (Anonymous, ISO 10705-2, 2001; Anonymous, ISO 10705-1, 2001; WHO, 2017) and the European Commission included somatic coliphages for the revised EU drinking water directive (2020), highlighting the importance of the method for understanding the risk profile of different treatment works. In addition, coliphage could be used to assess viral water quality impacts due to natural (i.e., runoff) or anthropogenic (i.e., sewer overflows) forces. An approach to appraise the risk is based on source water viral risk characterization, e.g., the presence of somatic coliphage exceeding 50 PFU/100 ml raw water would trigger the use of this microbial water quality parameter. This will be analyzed interstage in the water treatment works to demonstrate log removal to ensure that the risk of a breakthrough of pathogenic viruses is controlled. A meta-analysis revealed that the total coliphage median density in untreated wastewater was about 80,000 PFU/100 ml compared to around 30 PFU/100 ml for lowland river source waters used for drinking water sources (Nappier et al., 2019). The justification for use of somatic coliphage is that it is the most readily detected and at greater density justifying its selection as a useful indicator of viral risk (Jebri et al., 2017). Coliphages have been readily employed for log removal credit and assessing viral risk from raw water quality changes and transient operational or process events within water treatment works (Figures 1B,C). It is noteworthy to mention that the total coliphage concentrations do not always correlate to the total concentrations of infectious viruses whose abundance is ephemeral and often linked to outbreaks within catchments. However, the presence of specific indicators (e.g., coliphages) has been linked to viral water quality and likelihood of the presence of human enteric viruses.

## Tools for monitoring phage and viral transience and loading

Emergent technologies may facilitate detection of coliphages using molecular methods (PCR-based), direct enumeration using optical properties or fluorescent dyes, and indirect methods using enzymatic or immunological reactions. Among these, PCR-based methods have been applied extensively to different water matrices for coliphage detection. Primer sets have been developed for detecting type strains representing the families in somatic or F^+^ coliphages (Table 1). Real-time PCR has been used for the four somatic coliphage families, represented by type strain T4 for *Myoviridae*, type strain phiX174 for *Microviridae*, and type strain lambda (λ) phage for *Siphoviridae* and type strain T7 for *Podoviridae* (Lee, 2009). Despite the initial categorization of the genogroups of FRNAPH based on serological and physicochemical properties, development of genogroup-specific primers enables a more specific detection of the FRNAPH genogroups (Ogorzaly and Gantzer, 2006). Molecular methods such as PCR and sequencing are fast and reliable but complex and costly especially for routine water quality assessment. One alternative is the adenosine triphosphate (ATP) assessment, which is a rapid, sensitive, and is amenable to automated inline/real-time sensing of the microbiological activity in water samples (Ochromowicz and Hoekstra, 2005; Vang et al., 2014). Continuous sampling combined with ATP measurements displays potential for microbial drinking water quality assessments. The ability of the ATP assay to detect microbial ingress or poor biostability is influenced by both the ATP load from the contaminant event and the ATP concentration in the specific drinking waters (Vang et al., 2014). While ATP is an indicator of total microbial activity, its application or direct correlations with coliphage concentrations is not reported. This raises the prospect of whether online technologies (such as online ATP or particle count) could better inform/trigger coliphage sampling campaigns to assess pathways for health risks.

Another approach is direct quantification and this method relies on virus and their genomes being labeled using either specific or non-specific fluorescent dyes which permits quantification of virus-like particles (VLP) and viruses in environmental water samples using quantification tools, e.g., flow cytometry (Figure 1C) (Gaudin and Barteneva, 2015; Huang et al., 2016; Safford and Bischel, 2019; Olivenza et al., 2020). The tools have been adapted and applied to characterize phages including coliphages (Wilhartitz et al., 2013; Roudnew et al., 2014). For example, imaging flow cytometry was used to quantify somatic coliphages phiX174 and *E. coli* phage Qβ were detected with high specificity (within a concentration range of 10^4^-10^7^ PFU/ml) providing results in 1 h with results comparable to the double-layer agar technique (Yang et al., 2019). Rajnovic and Mas (2020) evaluated the sensitivity of a resazurin-reporter sensor in *E. coli* DSMZ 613/T4 phage model using a fluorimeter. Viable bacterial host cells reduce resazurin to a fluorescent compound resorufin which can be enumerated and phage-induced changes of activity of the bacterial host are measured using resazurin as a reporter. Green fluorescent protein (gfp)-based bacterial tagging used epigenetic biosensing to provide sensitive detection of infectious liposaccharide-binding phages (1.6 phages per 100 ml) (Olivenza et al., 2020). Other fluorescent tracer compounds have been developed to demonstrate log removal values (LRV) and have been applied in different scenarios. An example is TRASAR^®^, an organic fluorescent dye that carries a net negative charge, thus mimicking some of the charge repulsion experienced by negatively charged virus particles and can be detected at level of 10 μg/L with proprietary online sensor. LRVs for MS2 coliphage, TRASAR^®^, and conductivity using intact membranes averaged 6.2, 4.3, and 1.7, respectively, suggesting the tracer compound acts as a conservative surrogate of MS2 LRVs (Steinle-Darling et al., 2016). Nanoparticles or nanoplastics (tagged and/or coated) have been readily applied in assess fate and transport studies of virus in bench of lab scale sand columns. Good association between nanoparticles and two pathogenic virus suggested that physical tracers could be employed in fundamental and applied studies of virus or phage (Pang et al., 2014). Applicability of fluorescent dyes or nanoparticles to represent viruses or phages in environmental matrices requires further investigation especially bench scale or pilot studies to better understand the behavior and removal in treatment processes (Pulido-Reyes et al., 2022).

## Online monitoring tools: Limitations, future outlook, and recommendations

There are several limitations associated with detection of phages using fluorescence-based technologies, particularly in real-environmental water matrices. The detection limit of flow cytometry is ~150 kbp (Dlusskaya et al., 2021), whereas majority of bacteriophages (87%) with known genomes in NCBI database have a smaller genome size than this. Therefore, most bacteriophages and small enteric viruses can not be directly quantified using flow cytometry with the current limits of sensitivity—assuming the phage will be planktonic and not particle associated. Pathogenic viruses are also present and can pose health risks at concentrations well below the detection limits of 100 pathogens/ml, and as such the direct FCM-based assessment of waterborne pathogens is challenging (Ramírez-Castillo et al., 2015). Coliphages, however, are suitable process indicators and surrogates of human viral pathogens present at higher concentrations but are labor intensive to measure. We posit that there could be a plausible link between data generated from online tools, e.g., turbidity, online flow cytometry, or fluorescence (e.g., tryptophan-like), a strategy to understand the removal performance for virus and their indicators (Figure 1; Rockey et al., 2019). Biosensors offer another approach for monitoring of contaminants/indicators in water matrices (Gautam et al., 2012). Biosensor development using FACS, flow cytometry, imaging cytometry, OD-based techniques, resazurin reduction, gfp tagging, or ATP measurements is a promising technology. However, a thorough investigation of the matrix effects from environmental matrices on phage detection techniques is warranted. To date, application of “online” FCM has not been demonstrated for automated, real-time detection of waterborne viruses and requires further development (Safford and Bischel, 2019).

There are other online monitors such as turbidity and particle-size distribution which are being used in a diagnostic capacity by drinking water treatment plants (DWTPs) operators for monitoring raw and treated water quality. Interesting applications showed that elevated water flow levels linked to rainfall or snowmelt events increased the viral and phage concentrations in a catchment (Fauvel et al., 2016; Sylvestre et al., 2021). Infectious F-specific RNA bacteriophage concentrations increased during rainfall events (Fauvel et al., 2016), whereas viral concentrations increased by ~0.5-log during two snowmelt/rainfall episodes and ~1.0-log following a planned wastewater discharge upstream of the drinking water intake (Sylvestre et al., 2021). Another approach is to utilize enzyme-based sensors specific to coliforms. These provide rapid sensitive online monitoring, providing a proxy for culture-based assays. Online enzyme sensors such as β-D-glucuronidase provided estimates of increased viral loads, for example, transient peaks in raw water fecal contamination were identified at two urban DWTPs (Sylvestre et al., 2021). However, the enzyme sensors monitor bacterial indicators, and although their application for viral or phage quantification may not be accurate, the automated enzymatic sensors could be practically used to trigger intensive sampling campaigns. Catchment-level demographic and hydroclimatic parameters (agricultural or residential catchment, sediment property, precipitation frequency, and load) drive the physico-chemical properties such as turbidity, particulate, and water flow, and thereby affect the coliphage detection. Moreover, inhibitors present in water matrices are varied and impact the LOD achieved during enumeration. Hence, standardization of the enzyme sensors in different catchments is recommended prior to the implementation of monitoring program.

Focus to develop the fluorescent detection methods and their assessment criteria for coliphage detection offers opportunities to setup a reliable online monitoring system. We recommend matrix-based rigorous background controls for quantitative evidence of the sensitivity and accuracy as applied to aquatic environmental matrices and standardization of biosensing methods using representative coliphages [somatic, phiX174, and F-specific coliphage (MS2)]. There is an additional need to validate that potential virus and phage populations identified are indeed viruses rather than bacterial debris or other small particles. To achieve this, development of reliable positive controls, and internal standards is needed through evaluations using mixture of fluorescent-labeled bacteriophages or VLP of various sizes, genomes, and capsid characteristics. Method standardization is a herculean task that needs to be undertaken to take fluorescent methods to the next stage of real-time coliphage monitoring. If realized, this will help operators optimize DWTP for viral removal and assess potential pathways for risks for public health.

## Conclusions

- Coliphages have a host range broader than *E. coli* and have highly variable surface characteristics which govern partitioning in aquatics systems and influence removal during water treatment.
- General coliphage groups, either somatic coliphages or F^+^ coliphages, are valuable for investigating the virus elimination of water treatment.
- Current poor process level understanding of phage particle interactions, phage reproduction cycles within source water, and water treatment combined with methodological challenges is limiting the utility of coliphage as a sanitary tool in aquatic environments.
- The combined use of chemical and biological analyses alongside inline sensors could enable phage and thereby virus monitoring and enable the revision and development of more accurate environmental risk assessment.
- Finally, standardization of rapid online monitoring tools (e.g., turbidity and flow cytometry) for coliphages is recommended as a future area of activity.

## Author contributions

SS, RP, and FH wrote the manuscript and undertook the literature survey. All authors had substantial contributions to the conception and design of the work and approved the final version to be published.

## Funding

This work was funded by the UK Water Industry Research Limited under funding for the Independent Microbial Advisory Service at WRc and Cranfield University.

## Conflict of interest

The Water Research Centre received funding from the United Kingdom Water Industry Research. The funder was not involved in the study design, collection, analysis, interpretation of data, the writing of this article, and the decision to submit it for publication. The authors declare that the research was conducted in the absence of any commercial or financial relationships that could be construed as a potential conflict of interest.

## Publisher's note

All claims expressed in this article are solely those of the authors and do not necessarily represent those of their affiliated organizations, or those of the publisher, the editors and the reviewers. Any product that may be evaluated in this article, or claim that may be made by its manufacturer, is not guaranteed or endorsed by the publisher.
